# CPD: Test yourself

**Published:** 2011-09

These continuing professional development (CPD) Test Yourself questions are based on the contents of this issue. You can use the questions to test your own understanding; we hope that you will also discuss them with your colleagues and other members of the eye care team. The questions have been developed in association with the International Council of Ophthalmology (ICO) and are based on the style of the ICO Advanced Examination: **www.icoexams.org/exams/advanced**

**Table:** 

**1**.	**How can we prevent people from developing diabetic retinopathy?**	**True**	**False**
a	Type 1 diabetes is preventable	□	□
b	The risk of Type 2 diabetes can be reduced by exercise and a healthy diet	□	□
c	Good control of blood pressure reduces the risk of developing retinopathy	□	□
d	The newest blood pressure drugs are better for prevention of diabetic retinopathy than older, cheaper medicines	□	□
**2**.	**What should we tell people with diabetes about diabetic retinopathy?**	**True**	**False**
a	If you control your blood sugar, you will never get retinopathy	□	□
b	If you have diabetes, you will go blind because there is no effective treatment for diabetic retinopathy	□	□
c	You cannot tell whether or not you have retinopathy, and should be examined every year to detect it at an early stage	□	□
d	It is unreasonable for you to be upset about the long waiting times in our clinic — having your eyes checked should be your first priority	□	□
**3**.	**In a patient with diabetic maculopathy, which of the following are true?**	**True**	**False**
a	If there are exudates within 1 disc diameter of the fovea, there is a risk of losing vision	□	□
b	There is no treatment for ischaemic maculopathy	□	□
c	All patients with treatable maculopathy will have reduced vision	□	□
d	Intravitreal steroid injection is the best treatment for diabetic macular oedema	□	□
**4**.	**When planning a programme for diabetic retinopathy, which of the following are true?**	**True**	**False**
a	All patients with diabetes should have their retinas examined every year	□	□
b	Laser treatment should be available for everyone who needs it	□	□
c	Only ophthalmologists can detect diabetic retinopathy	□	□
d	Diabetes is only found in rich people who live in cities	□	□

## ANSWERS

1. **a. False**. Type 1 diabetes is caused by auto-immune destruction of the insulin secreting cells in the pancreas and cannot be prevented, **b. True. c. True. d. False**. There is no evidence that any drug is superior to any other. The important thing is to lower the blood pressure.
2. **a. False**. Perfect control is unobtainable. Good control reduces the risk of DR and delays it, but most people with diabetes will eventually get some DR. **b. False**. Very few people will go blind from DR if they are treated. It is important to inform them of the risks, but not to frighten them away. They need to know that effective treatments are available, **c. True. d. False**. People with diabetes have lives, families, and jobs. We need to ensure that detecting and treating retinopathy is simple, quick, and inexpensive.
3. **a. True. b. True. c. False**. Patients who have oedema near the fovea may still have normal vision, although they need treatment, **d. False**. A clinical trial showed that intravitreal steroid was not as good as laser, and may cause glaucoma.
4. **a. True. b. True. c. False**. Non-ophthalmologists may be trained to detect retinopathy in photographs, **d. False**. Diabetes is more common in urban populations, but it is becoming much more common everywhere, including poor and rural communities.

## Diabetic retinopathy quiz

Classify these photographs according to the table on page 12 and say which patients must be referred to a retinal clinic.

**Figure:**
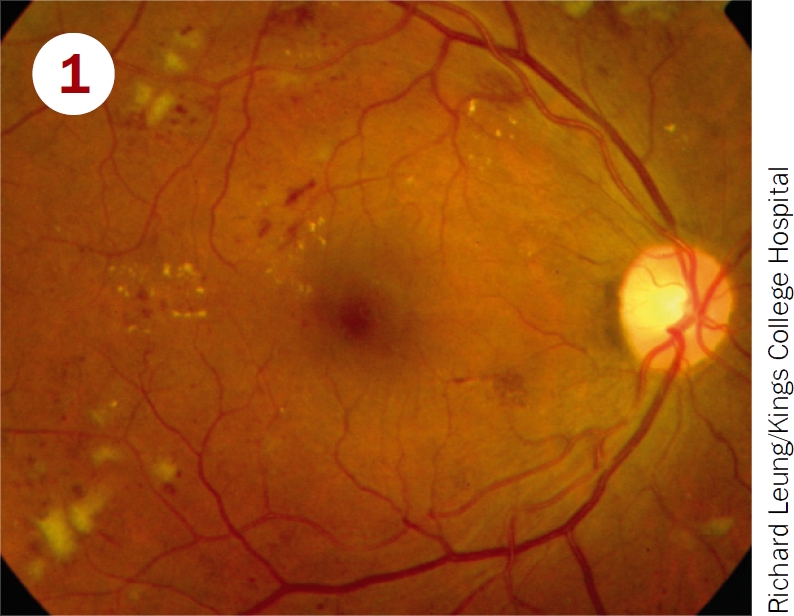


**Figure:**
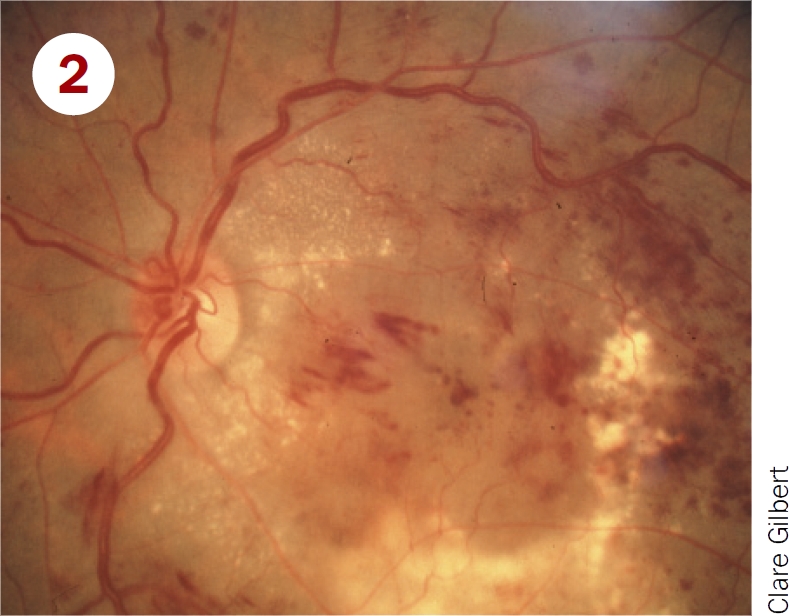


**Figure:**
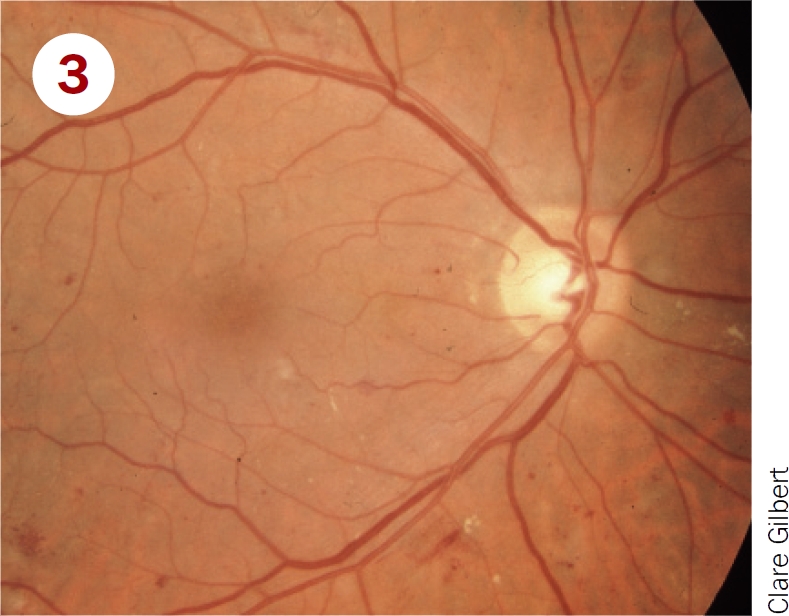


**Figure:**
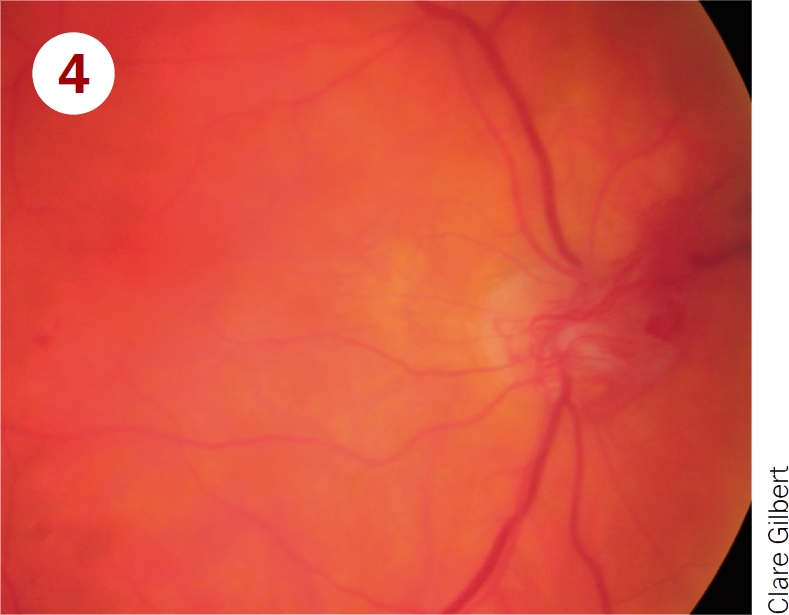


## ANSWERS

1. Moderate non-proliterative retinopathy, and maculopathy with exudates close to fovea. Should be referred for treatment of maculopathy.
2. Severe NPDR and maculopathy. Should be referred for treatment of maculopathy and watched closely for development of new vessels.
3. Mild non-proliferative retinopathy; pictures shows microaneurysms only. Should be examined again in 12 months.
4. Proliferative diabetic retinopathy; picture shows disc new vessels. Requires urgent referral for pan-retinal laser.

